# Impact of Display Sub-Pixel Arrays on Perceived Gloss and Transparency

**DOI:** 10.3390/jimaging10090221

**Published:** 2024-09-08

**Authors:** Midori Tanaka, Kosei Aketagawa, Takahiko Horiuchi

**Affiliations:** 1 Graduate School of Informatics, Chiba University, Yayoi-cho 1-33, Inage-ku, Chiba 263-8522, Japan; horiuchi@faculty.chiba-u.jp; 2 Graduate School of Science and Engineering, Chiba University, Yayoi-cho 1-33, Inage-ku, Chiba 263-8522, Japan; 24wm3210@student.gs.chiba-u.jp

**Keywords:** shitsukan, appearance, glossiness, transparency, sub-pixel array, display specification

## Abstract

In recent years, improvements in display image quality have made it easier to perceive rich object information, such as gloss and transparency, from images, known as shitsukan. Do the different display specifications in the world affect their appearance? Clarifying the effects of differences in pixel structure on shitsukan perception is necessary to realize shitsukan management for displays with different hardware structures, which has not been fully clarified before. In this study, we experimentally investigated the effects of display pixel arrays on the perception of glossiness and transparency. In a visual evaluation experiment, we investigated the effects of three types of sub-pixel arrays (RGB, RGBW, and PenTile) on the perception of glossiness and transparency using natural images. The results confirmed that sub-pixel arrays affect the appearance of glossiness and transparency. A general relationship of RGB > PenTile > RGBW for glossiness and RGB > RGBW > PenTile for transparency was found; however, detailed analysis, such as cluster analysis, confirmed that the relative superiority of these sub-pixel arrays may vary depending on the observer and image content.

## 1. Introduction

In image representation on a display, the number of pixels specifies the resolution, and the spatial resolution, which affects image sharpness, is an imperative factor in determining the appearance of an image [1]. In recent years, subpixel structures beyond the conventional red-green-blue (RGB) array have been widely adopted. These structures include variations such as RGB and white (RGBW), cyan (RGBC), and yellow (RGBY), as well as PenTile and other arrays. The ITU-R [2], the wireless communications arm of the International Telecommunication Union, recommends a standard viewing distance equivalent to 30 cycles per degree (cpd), which corresponds to the upper visual frequency limit for visual acuity of 20/20. This 30 cpd is the distance between two pixels at an angle of 1 min with the observer’s eyes. Resolution, defined by the number of pixels in a display, is the most critical factor in display image quality. Higher resolution means more pixels, resulting in a more detailed image. Resolution also significantly affects our visual information processing. Masaoka et al. [3] showed that increasing the angular resolution of an image from 30 cpd to 60 cpd enhances the perceived realness of objects in the image. However, the different pixel structures in newer displays, such as OLEDs, cause their perceived resolution to differ from that of conventional displays with the same resolution.

Baek et al. [4] showed that resolution differences can be discriminated in psychophysical experiments using modified Landolt C, and Zhan et al. [5] proposed an optical method using polarization multiplexing, reporting improved resolution in near-eye displays. These studies assumed conventional RGB sub-pixel array displays. Recently, displays with various sub-pixel arrays have been developed to enhance luminous efficiency and other factors. In our previous research, we focused on RGB sub-pixel arrays, RGBW sub-pixel arrays, and PenTile RGBG sub-pixel arrays, experimentally confirming that resolution differs depending on these sub-pixel arrays [6]. Additionally, we investigated the pixel aperture ratio and experimentally confirmed that resolution also varies based on the pixel aperture ratio [7]. 

Reports indicate that differences in pixel structures, such as subpixel array and pixel aperture ratio of displays, affect perceptual resolution. However, the aforementioned our studies primarily investigated the effects of these structural differences on perceptual resolution. The recent increase in display quality has made it easier to perceive glossy and transparent qualities in images, necessitating an investigation into how pixel structures affect not only perceptual resolution but also perceptual shitsukan. Studies have reported that image resolution affects the perception of shitsukan. Shishikui et al. [8] found that differences in angular resolution affect both lower-order impressions, such as perceptual resolution and color vividness, and higher-order impressions, such as realism, freshness, and beauty, using four images of food and plants. However, this study primarily focused on angular resolution conditions and did not investigate the appearance of shitsukan with different pixel structures.

Humans judge the quality and behavior of objects based on color and visual information [9]. Shitsukan, a Japanese term, describes the comprehensive sensations perceived from physical stimuli. Recent research on shitsukan has been active in various fields, such as information engineering, psychophysics, and neuroscience [10]. Our research has focused on shitsukan perception related to glossiness, translucency, and roughness, using real objects and images [11,12,13]. We have analyzed the relationship between perceived shitsukan and physical quantities obtained through psychophysical experiments. For example, Tanaka et al. [11] investigated shitsukan perception of real objects and images, analyzing resolution differences using 34 real samples of materials such as stone, paper, and glass, along with colorimetric reproduction images of high- and low-resolutions. Results revealed that perceived shitsukan qualities can differ between real objects and images with different resolutions. Consequently, we are developing shitsukan management technology to ensure consistent perceived shitsukan across displays with different specifications, such as resolution, and have proposed image processing methods based on human visual characteristics during shitsukan observation [14,15].

Clarifying the differences in pixel structure affect shitsukan perception, a previously unexplored area, is crucial for achieving effective shitsukan management across displays with varying pixel structures. This study therefore aims to experimentally investigate the impacts of display pixel arrangements on shitsukan perception. The visual evaluation experiment will use natural images to investigate the how three types of sub-pixel arrays (RGB, RGBW, and PenTile RGBG) influence the perception of glossiness and transparency.

## 2. Experiment

In this experiment, stimuli featuring RGB, RGBW, and PenTile RGBG (hereafter denoted as PenTile) sub-pixel arrays were created for 20 different natural images sourced from standard images and an open database [16]. The stimuli were presented on the left and right sides of the screen and observers’ visual evaluations of glossiness and transparency were used to investigate how different sub-pixel arrays affect the perceived shitsukan of the natural images shown.

### 2.1. Experimental Stimuli

**Figure 1:**
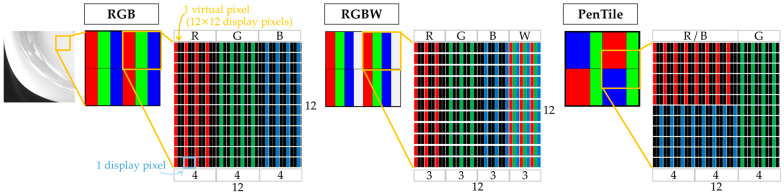
Construction of the virtual sub-pixel array for experimental stimuli.

We generated image stimuli using simulation methods from previous studies to prepare stimuli with different display sub-pixel arrays [6,7]. First, we describe the generation method. Given that the display used in the experiment (ColorEdge PROMINENCE CG3146, EIZO Corp., Ishikawa, Japan) has an RGB sub-pixel array, we treated 12 × 12 pixels of the actual display as one virtual pixel. The three sub-pixel array configurations used in the experiment—RGB, RGBW, and PenTile—are shown in Figure 1. To standardize the virtual pixel widths for each sub-pixel array, we set the virtual sub-pixel width as follows: 4 × 12 pixels for RGB, 3 × 12 pixels RGBW, and 8 × 12 pixels for the R and B sub-pixels and 4 × 12 pixels for the G sub-pixel in PenTile. To control conditions, the aperture ratio of the virtual sub-pixels was unified to 100% for all experimental stimuli. A spectroradiometer (CS-2000, Konica Minolta, Inc., Tokyo, Japan) was used to ensure that differences in luminance did not affect the evaluation by adjusting pixel values so that the average luminance of the experimental stimuli was equal across the three sub-pixel arrays. White was set to 334.2 cd/m^2^, ensuring the maximum luminance was consistent for each sub-pixel array. The 12 × 12 pixels of the experimental stimulus, created by this procedure, were considered as one virtual pixel. Consequently, evaluating the experimental stimuli with three different sub-pixel arrays was possible by observing one virtual pixel from a viewing distance 12 times that of one real pixel. 

**Figure 2:**
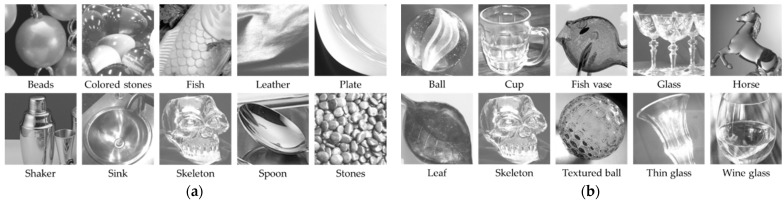
List of image content used as experimental stimuli. (**a**) Glossiness. (**b**) Transparency.

Next, we describe the content of the image stimuli. A total of 20 natural images were used, consisting of 10 glossy and 10 transparent images selected from standard images (SCID, SHIPP) and the Flickr Material Database [16]. The images were cropped to 150 × 150 pixels and grayscaled using the NTSC formula to eliminate the color influence on shitsukan perception. Experimental stimuli were prepared by enlarging one real pixel of the cropped natural image (150 × 150 pixels) by a factor of 12 × 12, resulting in image stimuli of 1800 × 1800 pixels (150 × 150 virtual pixels) with actual display pixels. Figure 2 illustrates the experimental stimuli for both the 10 glossy transparent evaluations. The glossiness stimuli featured various types of gloss based on Hunter’s six types of gloss [17], while the transparency stimuli included clearly transparent glass objects.

### 2.2. Experimental Procedure

Stimulus pairs simulating different sub-pixel arrays were presented on the left and right sides of a black background of a display, and observers were asked to rate which stimulus they felt more shitsukan (glossiness or transparent) using a two-alternative forced-choice (2AFC) test. A total of six pairs of experimental stimuli were used in a total combination of three different sub-pixel arrays. The stimulus pairs comprised image stimuli with the same content but in different pixel arrays. 

In the experiment, observers initially completed a visual acuity test. They then adapted to the lighting conditions in a darkened room by viewing a display with a black background for 3 min. After this adaptation period, stimulus pairs were presented on the left and right sides of the display. Participants used a 2AFC method to select the stimulus they perceived as having the stronger shitsukan for each pair. Following each response, a black background was presented for 1 s to eliminate the influence of the previously evaluated stimulus. Then, the next stimulus pair was presented, and the evaluation process was repeated. The stimuli were displayed from a viewing distance that was 12 times the size of a single pixel, simulating three different sub-pixel arrays. The distance was set to the equivalent of 30 cpd (7.01 m), in line with the standard viewing distance recommended by the ITU-R. At the distance, with 20/20 visual acuity, the pixel arrays are not discernible. Each pair was evaluated 8 times to ensure reliability. To counteract any potential effects from uneven display luminance, the stimulus pairs were presented with left-right swaps. Overall, each participant completed 480 evaluations (6 pairs × 8 evaluations per pair × 10 different image contents). The order of stimulus presentation was randomized for each shitsukan image to minimize bias.

## 3. Results and Discussions

In this section, the responses obtained in the evaluation experiment are summarized as experimental results for each shitsukan evaluated. Eleven observers with binocular acuity equivalent to 20/20 and normal color vision participated in the experiment.

### 3.1. MTF Calculation

To consider differences in physical conditions such as sub-pixel array, we calculated the modulation transfer function (MTF), which serves as an evaluation index for the physical resolving power of an optical system [18], and compared it with the evaluation results. A higher MTF is expected to indicate clearer image display and, therefore, a higher perception of shitsukan.

**Figure 3:**
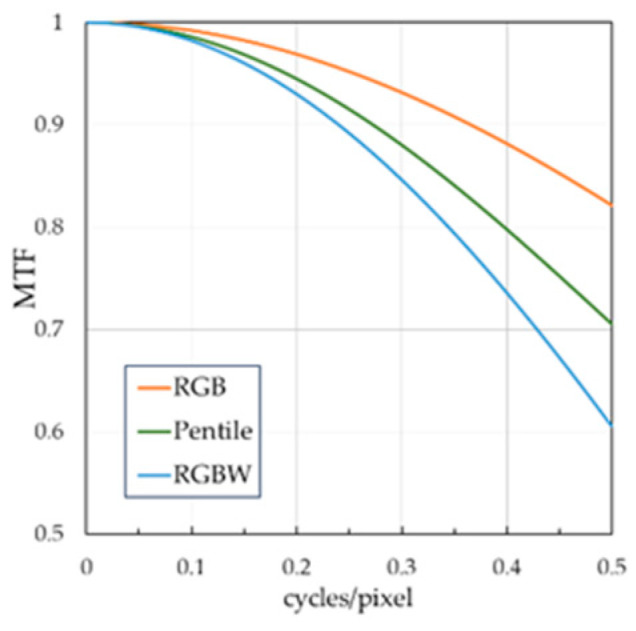
Calculated MTF for each sub-pixel array.

Figure 3 illustrates a graph of the calculated MTFs for the three sub-pixel arrays used in the experiment. According to the MTF values, the superiority relationship among the sub-pixel arrays was RGB > PenTile > RGBW. At the Nyquist spatial frequency of 0.5 cycles/pixel, the MTF differences between the RGB and RGBW, RGBW and PenTile, and PenTile and RGB sub-pixel arrays were approximately 21.6%, 10.0%, and 11.6%, respectively.

### 3.2. Response Rate and Significant Differences

**Figure 4:**
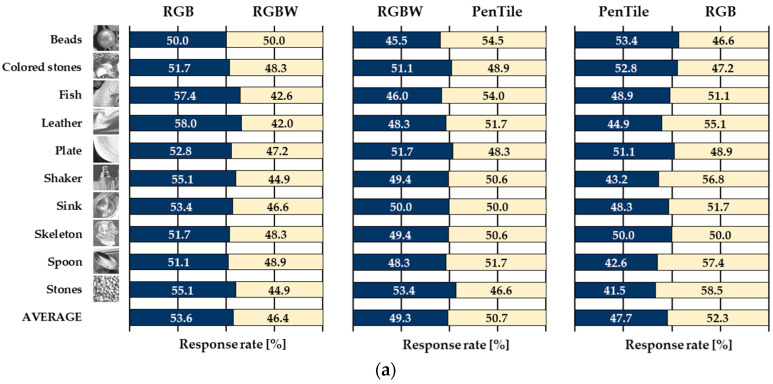

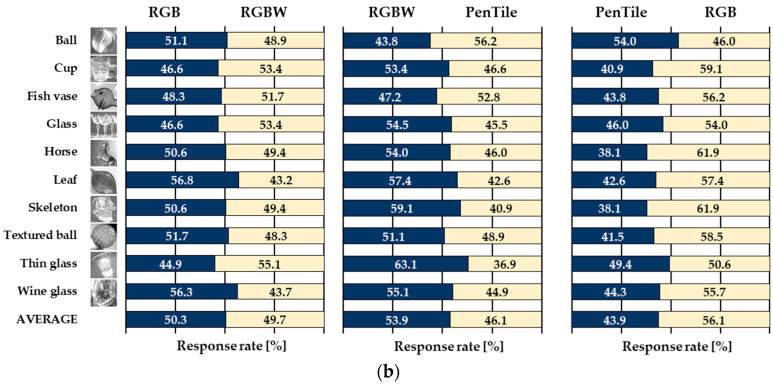
List of image contents used as experimental stimuli. (**a**) Glossiness. (**b**) Transparency.

The average response rate of the 11 respondents who answered which stimulus was more glossy/transparent for each experimental stimulus pair is shown in Figure 4. The *p*-values, standard deviations, and effect sizes and their indices, indicating whether there is a significant difference between the stimulus pairs of the corresponding sub-pixel array, are shown in Table 1. Cohen’s standard deviation was used to calculate the effect size to confirm whether there was a significant difference, and the closest index was selected with reference to Sawilowsky and Cohen for the determination of the effect size index [19,20]. The *p*-value, indicating significant differences between average response rates, revealed that the RGB sub-pixel array scored significantly higher in the RGB-RGBW stimulus pair for glossiness, and the PenTile sub-pixel array scored significantly higher in the PenTile-RGB stimulus pair for transparency. Although the response rate varies by image, the overall trend indicates that these pairs are significantly different, even when effect sizes independent of sample size are considered. In addition, when examining the relative shitsukan relationships between stimulus pairs in terms of effect sizes, a general relationship of RGB > PenTile > RGBW (consistent with the MTF superiority relationship) for glossiness and RGB > RGBW > PenTile (opposite to the MTF superiority relationship between PenTile and RGBW) for transparency is found.

Next, we briefly present the results by observer. For example, observer #6 responded that RGBW was glossier for the RGBW-PenTile stimulus pair and RGB was glossier for the PenTile-RGB stimulus pair (effect size: huge). Observer #10, on the other hand, answered that PenTile was glossier for the RGBW-PenTile stimulus pair and PenTile was glossier for the PenTile-RGB stimulus pair (effect size: huge and large, respectively). For transparency, observer #5 responded that RGBW was more transparent for the RGBW-PenTile stimulus pair, and RGB was more transparent for the PenTile-RGB stimulus pair (effect size: huge). Conversely, observer #3 responded that PenTile was more transparent for the RGBW-PenTile stimulus pair and PenTile was more transparent for the PenTile-RGB stimulus pair (effect size: large and very large). Thus, it can be seen that there are individual differences in judgments of the superiority of shitsukan relationships among the sub-pixel arrays and that the overall average values may be canceling each other out. These results suggest that the difference in sub-pixel array affects the perception of glossiness and transparency; however, it is necessary to consider the evaluation bias that generates observers’ response tendencies, so the results considering the cluster analysis results are discussed later in this section.

### 3.3. Cluster Analysis

#### 3.3.1. Observer Classification

**Figure 5:**
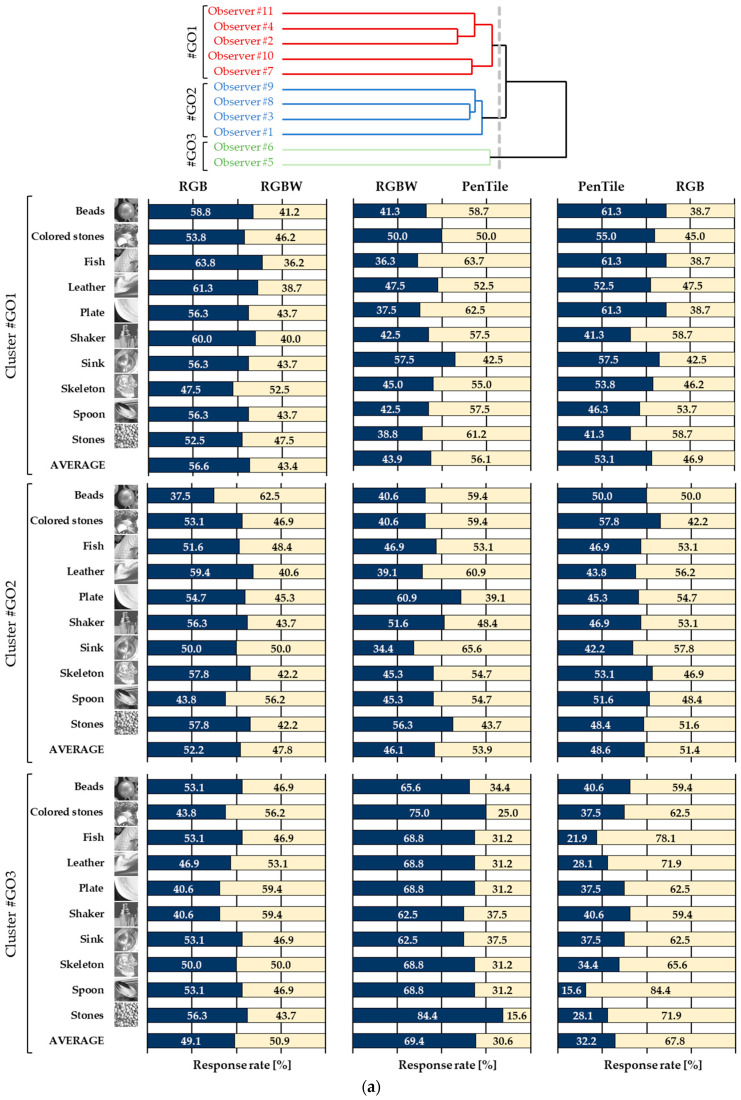

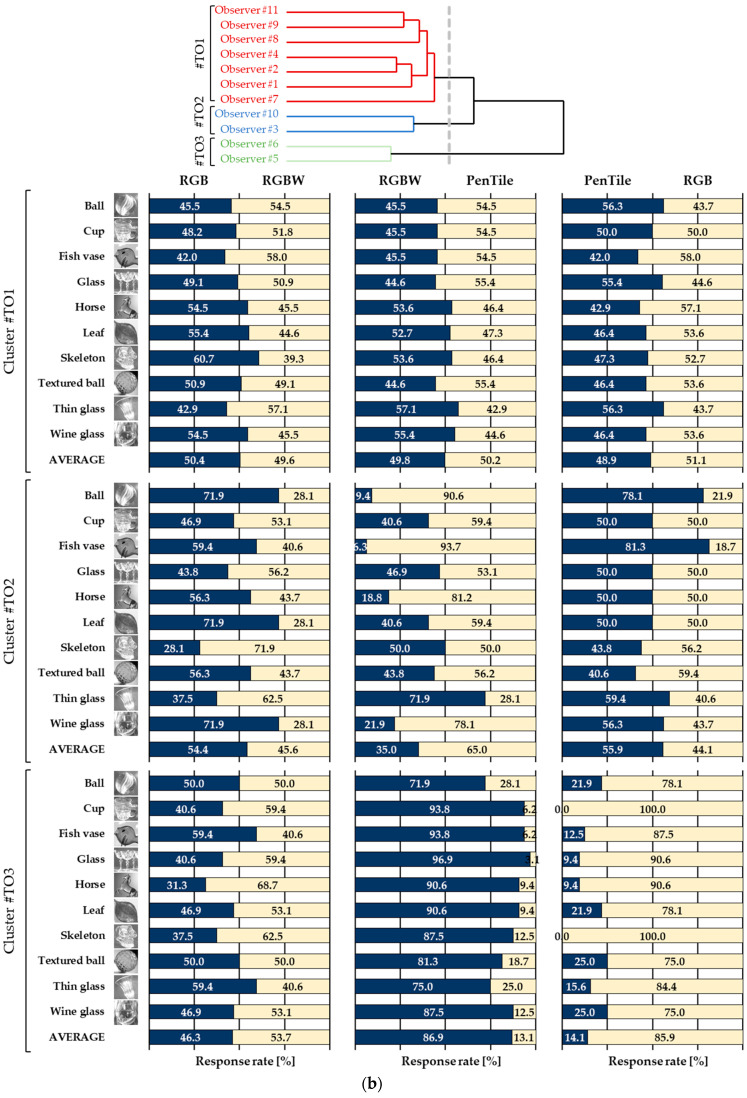
Dendrogram of observer cluster classification and corresponding response rates. (**a**) Glossiness. (**b**) Transparency.

Based on the evaluation experiment results, a classification of observers was performed through cluster analysis to account for individual response tendencies. Hierarchical clustering (Ward’s method) was used for this analysis. Table 2 and Figure 5 show the dendrogram of the observer clusters, the average response rate for each cluster (#GO1–GO3 for glossiness, #TO1–TO3 for transparency), and the significance indicated by effect sizes.

- Glossiness

The glossiness shown in Table 2 and Figure 5a can be classified into Cluster #GO1 (5 observers), Cluster #GO2 (4 observers), and Cluster #GO3 (2 observers). The response rates for each cluster confirm the relationships PenTile > RGB > RGBW for Cluster #GO1, RGB > PenTile > RGBW for Cluster #GO2, and RGBW > RGB > PenTile for Cluster #GO3. From Table 2, the significance of the superiority-inferiority relationships reveals that in Cluster #GO1, the difference between RGBW and other sub-pixel arrays is particularly large (effect size: huge), while in Cluster #GO3, the difference between PenTile and other sub-pixel arrays is also notably large (effect size: huge). Observers in Cluster #GO1 perceive glossiness as significantly lower with RGBW, while those in Cluster #GO3 perceive glossiness as significantly lower with PenTile. Conversely, Cluster #GO2 shows a milder effect size, indicating that the sub-pixel array has no significant effect on glossiness perception. However, response rates for stimuli such as Beads, Plate, and Sink in Cluster #GO2 exceed 60%, suggesting that the sub-pixel array does affect glossiness perception depending on the stimulus object.

- Transparency

The transparency shown in Table 2b and Figure 5b was categorized into Cluster #TO1 (7 observers), Cluster #TO2 (2 observers), and Cluster #TO3 (2 observers). The response rate for Cluster #TO1 indicates that there is a relationship of RGB > PenTile > RGBW, but because the effect size is small, observers in this cluster do not seem to be significantly affected by differences in the sub-pixel array in their perception of transparency. In Cluster #TO2, there is a relationship of PenTile > RGB > RGBW, and the difference between PenTile and other arrays (effect size: very large and large) is particularly perceived, as confirmed by the response rate and effect size. The response rate for each stimulus in Cluster #TO2 shows a number of response rates above the 75% general significance level for the two choices. However, some stimuli do not follow the average trend for the corresponding pair, such as Thin glass in the RGBW-PenTile pair (71.9–28.1%) and Skeleton in the RGB-RGBW pair (28.1–71.9%). Thus, the combination of image features and sub-pixel confirms that the appearance of transparency changes depending on the combination of image characteristics and sub-pixel array of the stimuli, and that it influences observers’ responses. Finally, the perceived transparency of Cluster #TO3 has a relationship of RGBW > RGB > PenTile, where the effect size is large and the *p*-value is significant, clearly indicating that the sub-pixel array has a significant effect on the perceived transparency. In terms of response rate details, Pentile was significantly less transparent for both image stimuli.

The results confirm that sub-pixel arrays influencing perceived glossiness and transparency varied by cluster, with notable trends: Clusters #TO2 (4 observers) and #TO1 (7 observers) perceived higher transparency in the order of RGB > PenTile > RGBW, consistent with the MTF superiority relationship, although the effect sizes were small.

Conversely, clusters with an ordinal relationship for glossiness and transparency differing from the MTF superiority relationship showed large effect sizes and clear criteria for shitsukan judgment, though they had fewer respondents. Detailed response rates revealed some deviations from the overall trend, with certain image stimuli affecting the perceived superiority of sub-pixel arrays. The next section will explore how specific image stimulus characteristics influence shitsukan appearance when comparing pairs with different sub-pixel arrays.

#### 3.3.2. Image Classification and Image Features

**Figure 6:**
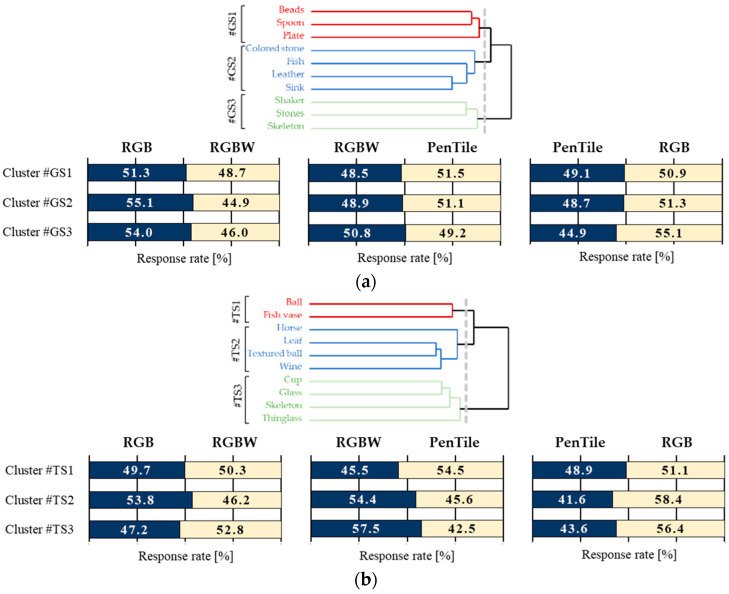
Dendrogram of stimulus cluster classification and average response rates for each stimulus cluster. (**a**) Glossiness. (**b**) Transparency.

**Figure 7:**
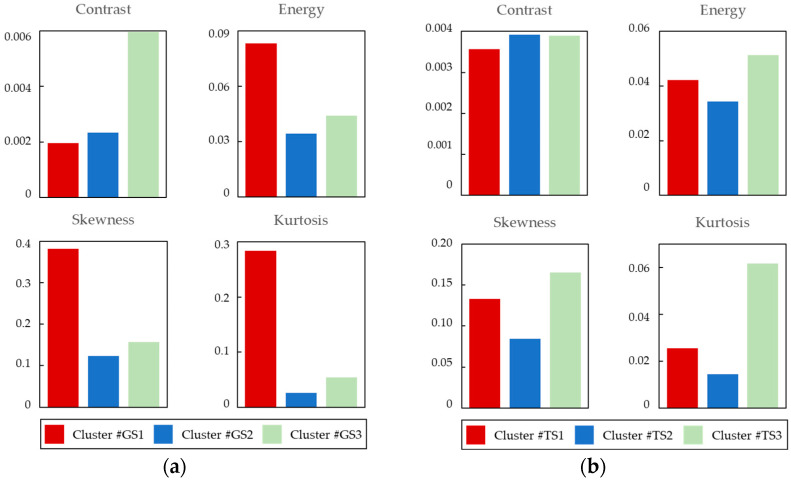
Image features for each stimulus cluster. (**a**) Glossiness. (**b**) Transparency.

In addition to differences in the sub-pixel array, experimental stimuli were classified by cluster analysis based on the response rate of each observer to investigate what image features influence the response tendencies of observers. Then, we calculated the image features of each cluster. The image features used are the Gray Level Co-occurrence Matrix (GLCM) [21], which is considered useful for texture analysis of objects in images, contrast (an index of local variation in luminance), energy (an index of texture repetition), and kurtosis and skewness calculated from the image histograms. Figure 6 illustrates the dendrogram of stimulus cluster classification and the average response rate for each stimulus cluster. Figure 7 illustrates the image features for each stimulus cluster, and Table 3 lists the effect sizes between stimulus pairs.

- Glossiness

For glossiness, the 10 stimuli were classified into three cluster based on observer response rates Cluster #GS1 (Beads, Spoon, Plate), #GS2 (Colored stone, Fish, Leather, Sink), and #GS3 (Shaker, Stones, Skeleton). The response rates and effect sizes confirmed a significant superiority relationship of RGB > PenTile > RGBW for Clusters #GS1 and #GS2 and RGB > RGBW > PenTile for Cluster #GS3.

Detailed examination of each cluster reveals that image stimuli in Cluster #GS1 exhibit global gloss spread over the entire smooth object surface. According to Hunter’s definition of gloss [17], these stimuli represent ‘distinctness-of-image (DOI) gloss’ or ‘absence-of-bloom gloss. Notably, dark regions, such as black, are widely distributed near the object’s gloss in these images, enhancing the object’s glossiness. As shown in Figure 7a, the values of Energy, Skewness, and Kurtosis for Cluster #GS1 are higher than those for other stimuli, indicating that these features contribute to a higher perception of glossiness, as reported in previous studies [22].

Cluster #GS2 includes four image stimuli: Colored stone, Fish, Leather, and Sink. These images characterized by both highlights and soft gloss on finely textured surfaces (e.g., the texture of Leather and the coarse-grained pattern of Sink), exhibit ‘Contras gloss’ and are classified as low gloss. The image features, including low skewness and kurtosis, indicate that these images are likely to be perceived as having low gloss. Clusters #GS1 and #GS2 both exhibit the same RGB > PenTile > RGBW sub-pixel array relationship as the MTF superiority relationship. Images with varying gloss levels—from low to high, including DOI gloss, presence-of-bloom gloss, and Contrast gloss—suggest that the RGB pixel array is optimal for expressing glossiness.

Cluster #GS3 includes three image types: Shaker, Stones, and Skeleton. These images, classified as medium gloss in the ‘Specular gloss’ category, represent the appearance of shininess and brilliance of highlights. The image features show high contrast with relatively low skewness and kurtosis. This indicates that the RGB sub-pixel array enhances the perceptibility of glossiness in images with high local luminance variability, i.e., images with glosses highlighted by local variations.

- Transparency

The 10 stimuli were categorized into Cluster #TS1 (Ball, Fish vase), #TS2 (Horse, Leaf, textured ball, Wine glass), and #TS3 (Cup, Glass, Skeleton, Thin glass) based on observer response rates. Based on the response rate and effect size, a superiority-inferiority relationship was confirmed for perceived transparency, RGB > PenTile > RGBW for Cluster #TS1, RGB > RGBW > PenTile for Cluster #TS2, and RGBW > RGB > PenTile for Cluster #TS3.

For image stimuli in Cluster #TS1, the transparency is uniformly distributed over a large area, featuring a mixture of dynamic and fine, crack-like patterns. The image features, illustrated in Figure 7b, indicate low contrast with other features located between Cluster #TS2 and TS3. This reflects the dynamic patterns of global luminance variations with low contrast, representing local variations. Although the response rate alone does not determine a clear superiority relationship for each sub-pixel array, the effect size analysis shows a significant relationship, with RGB > PenTile > RGBW, due to the huge effect size between PenTile and RGB.

For the four image stimuli in Cluster #TS2, the object surfaces exhibit fine patterns and finishes in addition to gloss. For example, the Wineglass image displays a fine pattern with subtle textures on both the object surface and transparent liquid. The high contrast, low energy, skewness, and kurtosis of the image features confirm that the object involves local luminance variations due to delicate patterns, combined with global dull luminance variations of a single color.

Finally, Cluster #TS3 contains four different image stimuli of glass object that create the impression of bright white transparency. In these stimuli, the luminance distribution is widely spread around the white light, giving a sense of transmitted light. For Clusters #TS2 and #TS3, the perceived transparency was significantly higher for RGBW compared to PenTile, despite MTF suggesting otherwise. This implies that a lower MTF, which is less pronounced at lower spatial frequencies, might enhance the perception of transparency by making the transmitted light appear more prominent.

These findings indicate that perceptions of glossiness and transparency vary significantly when the same image stimulus is displayed on screens with different sub-pixel arrays. Such differences cannot be fully explained by the MTF superiority-subordination relationship alone. This suggests that perceived glossiness and transparency depend on both the observer’s response tendencies and the image content. To manage shitsukan effectively across various display technologies, it is important to popularize methods that focus on MTF [23] while also accounting for shitsukan characteristics not explained by MTF alone. Developing shitsukan management technologies should involve considerations of human visual characteristics, observer evaluation biases, and the specific content of images.

## 4. Conclusions

This study experimentally investigated the effects of different display sub-pixel arrangements on shitsukan perception, an area previously unexplored, by comparing natural images with three sub-pixel arrangements (RGB, RGBW, and PenTile RGBG) through visual evaluation experiments. The findings revealed that, in general, RGB was perceived as superior to PenTile, which in turn was superior to RGBW for glossiness—a trend consistent with the MTF dominance relationship. For transparency, however, the trend was RGB > RGBW > PenTile, which contrasts with the MTF dominance relationship between PenTile and RGBW. Further analyses of clusters, effect sizes, and image features indicated that the perceived superiority of sub-pixel arrays can be influenced by observer biases and the nature of the image content. Specifically, images with varying levels of gloss, including DOI gloss, absence-of-bloom gloss, and contrast gloss, were perceived as glossier with the RGB sub-pixel array. The RGBW sub-pixel array demonstrated a significant increase in the perception of transparency compared to PenTile. These results underscore that sub-pixel arrangements impact the perception of glossiness and transparency. Future research should explore additional shitsukan attributes and pixel structures to further understand these effects.

These results suggest that sub-pixel array affects the appearance of glossiness and transparency. Further research is needed to explore these effects in greater detail and to investigate additional shitsukan attributes and pixel structures.

## Figures and Tables

**Table 1:** Effect sizes between stimulus pairs for all responses.

	(a) Glossiness			(b) Transparency		
	RGB-RGBW	RGBW-PenTile	PenTile-RGB	RGB-RGBW	RGBW-PenTile	PenTile-RGB
*p*-value	0.0020	0.4021	0.1321	0.7919	0.0567	0.0035
Std. Dev.	2.68	2.45	4.39	3.97	5.59	4.96
Effect size	2.71 Huge	0.56 Medium	1.05 Very large	0.17 Small	1.38 Very large	2.47 Huge

**Table 2:** Effect sizes between stimulus pairs for different observer clusters.

**(a) Gloss**.	**Cluster #GO1**			**Cluster #GO2**			**Cluster #GO3**		
**Stimulus Pair**	**RGB-** **RGBW**	**RGBW-** **PenTile**	**PenTile-** **RGB**	**RGB-** **RGBW**	**RGBW-** **PenTile**	**PenTile-** **RGB**	**RGB-** **RGBW**	**RGBW-** **PenTile**	**PenTile-** **RGB**
*p*-value	0.0015	0.0148	0.2308	0.3434	0.1652	0.3673	0.6164	0.0000	0.0001
Std. Dev.	4.68	6.44	7.84	6.92	8.18	4.68	5.71	6.39	8.47
Effect size	2.83 Huge	1.90 Huge	0.81 Large	0.63 Medium	0.96 Large	0.60 Medium	0.33 Small	6.07 Huge	4.21 Huge
**(b) Trans.**	**Cluster #TO1**			**Cluster #TO2**			**Cluster #TO3**		
**Stimulus Pair**	**RGB-** **RGBW**	**RGBW-** **PenTile**	**PenTile-** **RGB**	**RGB-** **RGBW**	**RGBW-** **PenTile**	**PenTile-** **RGB**	**RGB-** **RGBW**	**RGBW-** **PenTile**	**PenTile-** **RGB**
*p*-value	0.8545	0.9134	0.5414	0.3880	0.0460	0.2013	0.2229	0.0000	0.0000
Std. Dev.	5.99	5.05	5.34	15.25	20.51	13.62	9.06	8.31	9.46
Effect size	0.12 Small	0.07 Very small	0.40 Medium	0.57 Medium	1.46 Very large	0.87 Large	0.83 Large	8.88 Huge	7.60 Huge

**Table 3:** Effect sizes between stimulus pairs within each stimulus cluster.

	(a) Glossiness			(b) Transparency		
	RGB-RGBW	RGBW-PenTile	PenTile-RGB	RGB-RGBW	RGBW-PenTile	PenTile-RGB
*p*-value	0.0905	0.4639	0.2140	0.9142	0.5664	0.1336
Std. Dev.	1.94	1.22	2.33	3.37	6.27	3.74
Effect size	3.57 Huge	1.04 Very large	2.08 Huge	0.14 Small	0.79 Large	2.83 Huge

## Data Availability

Data available on request due to restrictions.
